# Total and Simultaneous Coverage Repair for the Obturator Hernia and Groin Hernias With One Large Mesh: A Case Report

**DOI:** 10.7759/cureus.69917

**Published:** 2024-09-22

**Authors:** Masaaki Urade, Shogo Maruzen, Hirofumi Terakawa

**Affiliations:** 1 General Surgery, Nanto Municipal Hospital, Nanto City, JPN; 2 Gastrointestinal Surgery/Breast Surgery, Kanazawa University, Kanazawa, JPN

**Keywords:** hernia reduction, incarceration, mesh, obturator hernias, transabdominal preperitoneal repair

## Abstract

Obturator hernia is a rare condition. Preoperative diagnosis is difficult to achieve because the hernia swelling is rarely palpable. Diagnosis is often delayed, and the hernia can become fatal if intestinal strangulation occurs, especially in older patients. Hesitation in the intervention will result in perforation, peritonitis, sepsis, and death. We herein report a case involving a Japanese woman in her 90s who visited our emergency room with nausea and right inner thigh pain. Computed tomography (CT) at onset revealed intestinal incarceration between the piriformis and external obturator muscles; therefore, a right-sided obturator hernia was diagnosed. Manual release of the incarceration, combined with echo probe manipulation and lower extremity movement, was successfully performed. The patient’s pain was dramatically reduced, and emergency surgery was avoided. A prompt hernia release after reaching the correct diagnosis is very important for obturator hernia patients. Scheduled minimally invasive surgery (transabdominal pre-peritoneal repair, TAPP) was subsequently performed. Intraoperatively, a coexistence of ipsilateral femoral hernia was detected by laparoscope. Therefore, we tried to cover not only the obturator canal but also the subclinical coexistence of ipsilateral groin hernias. All four hernia orifices (obturator hernia orifice, internal inguinal hernia orifice, external inguinal hernia orifice, and femoral hernia orifice) were covered at the same time with a single large mesh of 15 × 10 cm. Reports detailing such approaches (total and simultaneous coverage of the obturator canal and myopectineal orifice with one rectangular mesh) are relatively rare in the literature.

## Introduction

Patients with obturator hernia often present to the hospital with an acute abdomen without a history of laparotomy. What is the cause of acute abdomen other than intestinal adhesion? Clinical suspicion for hernias is very important in the diagnosis of acute abdomen. Among the many types of abdominal hernias, obturator hernia should be highly suspected whenever an elderly, thin, and multiparous female with no previous abdominal surgery develops small bowel obstruction. Hesitation in surgical intervention will result in serious consequences, such as perforation, pan-peritonitis, sepsis, and death [1].

Historically, the resection of the necrotic intestine was sometimes required because the absence of palpable swelling (a bulge or lump) made early and exact diagnosis very difficult. In the current era, computed tomography (CT) is the gold standard for definitive diagnosis. Prompt and precise diagnosis by CT scan and non-invasive manual release of incarceration can avoid the need for emergency laparotomy and bowel resection. Patients are usually asymptomatic when the intestine is not present within the obturator canal. Occasionally, however, when the intestine is within the canal, patients will experience a type of pain from the medial thigh to the hip joint termed “obturator neuralgia” [2]. These patients with refractory intermittent thigh pain but no abdominal symptoms might visit an orthopedist rather than a general surgeon. Some patients with vague complaints may be referred to psychiatry.

Obturator hernias are concurrently associated with other groin hernias, such as inguinal hernias and femoral hernias [3]. We herein present a successfully treated case with an incarcerated obturator hernia with a coexistent ipsilateral femoral hernia that was repaired by tension-free mesh herniorrhaphy using the transabdominal pre-peritoneal repair (TAPP) method [4]. A mesh is an artificial prosthesis that patches the hernia orifice and prevents bowel entry into the orifice. One large rectangular mesh was used to cover not only the obturator hernia orifice but also three other groin hernia orifices (internal inguinal hernia orifice, external inguinal hernia orifice, and femoral hernia orifice) at the same time. Total and simultaneous patching of the obturator canal and myopectineal orifice with a single large mesh represents an ingenious approach to obturator hernia repair. This minimally invasive laparoscopic method is an effective treatment for older patients and those who are considered poor candidates for surgical intervention.

## Case presentation

A 90-year-old woman visited our emergency room with lower abdominal pain, nausea, and right medial thigh pain that had developed a few hours before her arrival. Her medical history included hypertension and diabetes mellitus. Her reproductive history (TPAL score) was T3 P0 A0 L3: term birth, 3; preterm birth, 0; abortion, 0; living children, 3. She had no history of laparotomy. At presentation, she was 150 cm tall and weighed 38 kg. She was emaciated with a body mass index of 16.9 kg/m^2^. Physical examination revealed no swelling or skin lesions (ecchymosis or erosion) at the right thigh. The abdomen showed slight distension without signs of peritoneal irritation.

**Figure 1:**
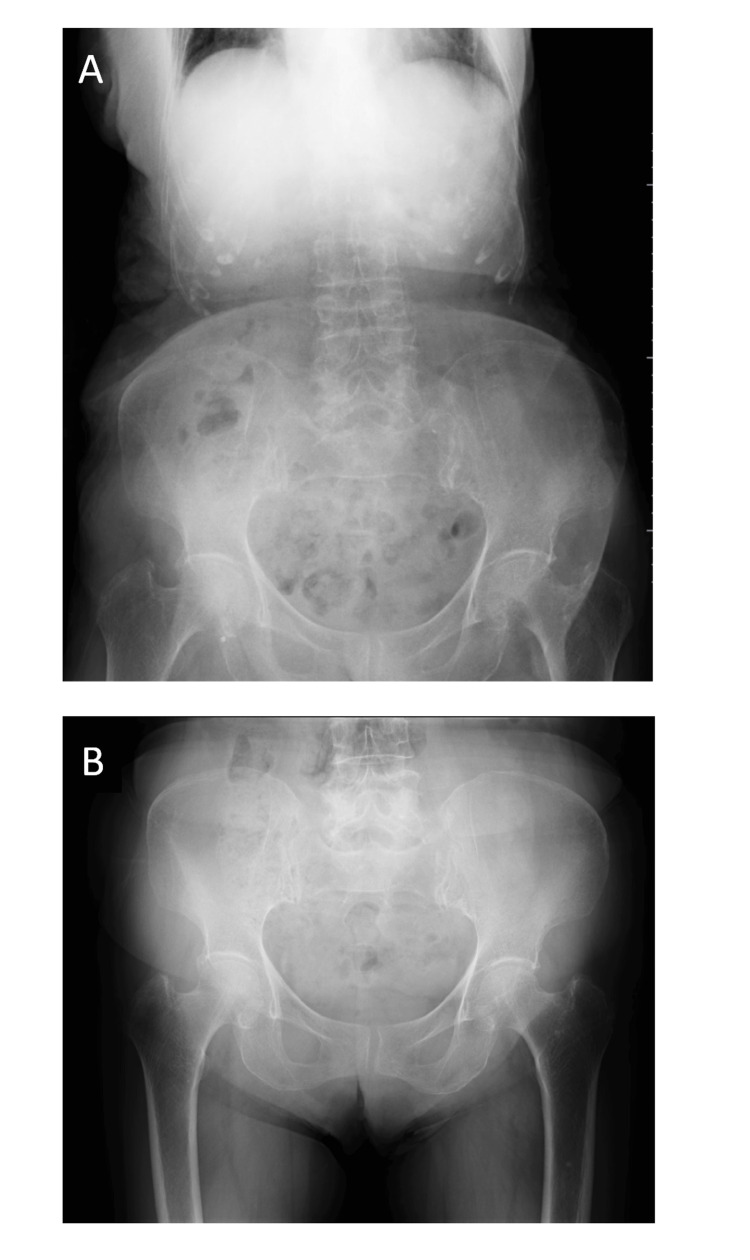
Abdominal roentgenogram and pelvic radiograph before admission (A) An abdominal roentgenogram in the supine position showing no dilated small intestinal loops and no abnormal intestinal gas. The degree of intestinal dilatation was slight because she had just developed ileus a few hours earlier. (B) The pelvic radiograph showing no abnormalities of the bones or joints in spite of her right thigh pain.

An abdominal X-ray before admission showed neither intestinal dilatation nor abnormal gas (Figure 1A). A pelvic X-ray showed no fractures of the right femur, sit bones, or pubic bones in spite of her right thigh pain (Figure 1B).

**Figure 2:**
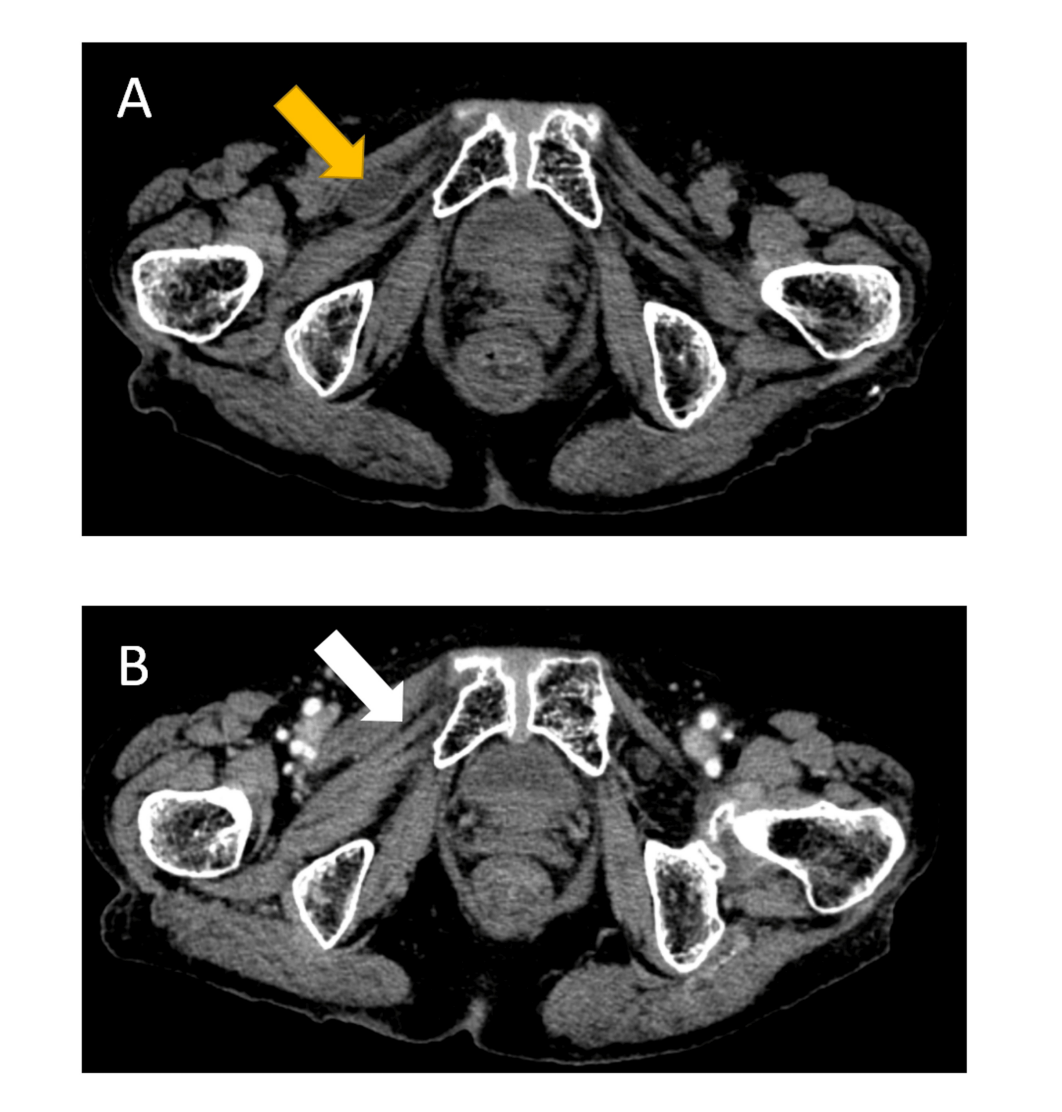
The first pelvic CT (at the onset) and the second pelvic CT (after the release of incarceration) (A) The first plain CT scan clearly demonstrated an obturator hernia presenting as a low-density round mass (yellow arrow) between the pectineus muscle and the external obturator muscle. (B) The second CT scan showed fine alleviation of intestinal packing from the obturator canal. Disappearance of a round mass (white arrow) between the two muscles was demonstrated. No intestinal ischemia was detected using a contrast medium.

The emergency room physician (a gastroenterologist) suspected intestinal obstruction, and CT scan was performed. The image obtained from plain pelvic CT revealed the incarceration of the intestine to the obturator canal between the external obturator muscle and the piriformis muscle (Figure 2A).

Therefore, the primary diagnosis was the right obturator hernia with the incarceration of the small intestine. The degree of intestinal dilatation was slight because she had just developed ileus a few hours earlier. The CT scan revealed no other subclinical hernias, such as femoral or inguinal hernia. The incarcerated intestine was pushed back while observing with the echo probe, and the patient’s right thigh pain and nausea were mitigated. On the same day, a second pelvic CT scan with enhancement revealed relief of the incarceration (Figure 2B), and no intestinal ischemia was detected.

After the release of hernia incarceration, watchful observation in the ward revealed no development of peritonitis secondary to perforation; therefore, we concluded that the relieved bowel was not at risk of necrosis. Re-incarceration did not occur before elective surgery. The patient’s hematological data were within the reference ranges except for glycated hemoglobin, which was 7.1%. The C-reactive protein concentration was 0.04 mg/dL, and the white blood cell count was 6,210 cells/μL with a neutrophil proportion of 61%. There were no elevations of the creatine phosphokinase, D-dimer, and lactate concentrations. Electrocardiography and respiratory function tests showed no relevant abnormalities. She did not develop systemic inflammatory response syndrome. 

**Figure 3:**
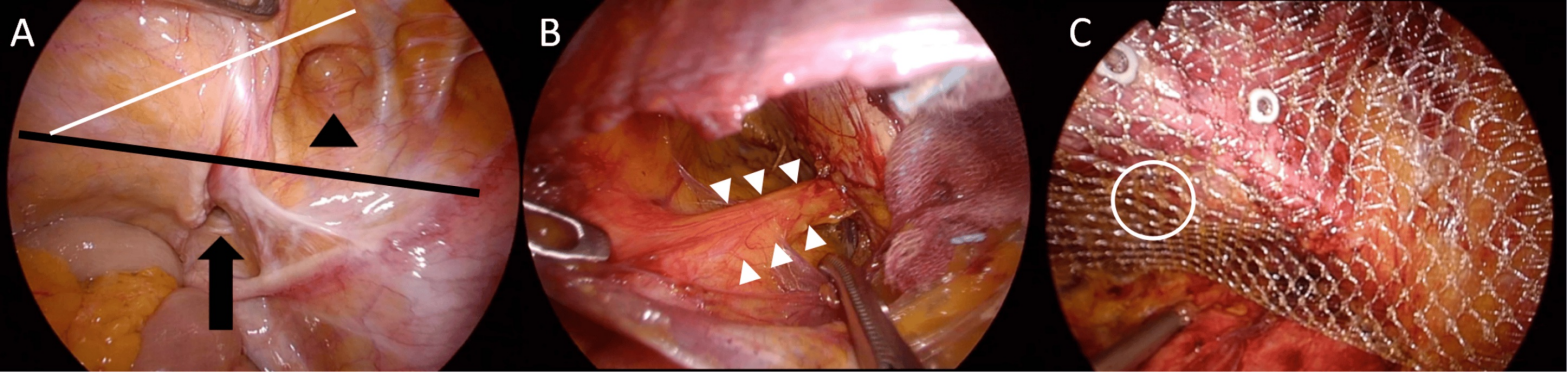
Intraoperative laparoscopic findings (A) The right obturator canal was expanded (black arrow), and the coexistence of an ipsilateral femoral hernia was detected (black arrowhead). (B) The preperitoneal fat plug (white arrowheads) was teased out from the obturator canal, keeping the obturator nerve intact. (C) Metal spiral tackers (seven shots in total) were used to fix the rectangular mesh. Three of the seven shots were made into the pubic bone (Cooper’s ligament). One shot was made into the pubic tubercle near the midline, and the other two shots were made near the obturator canal orifice (white circle) in order to cover the very hernia orifice surely and firmly. The thick black line in (A) is the right Cooper’s ligament (the pubic bone). The thin white line in (A) is the right iliopubic tract.

Three days after the temporal manual release, elective laparoscopic repair was safely performed. The obturator hernia surgery was performed using almost the same method used for groin hernia surgery. TAPP was performed. A 12-mm skin incision was made in the navel, and the first port was inserted for laparoscopy. Careful laparoscopic observation revealed no necrotic or reddish intestine and no ascites or adhesion. The right obturator canal was expanded, and the subclinical coexistence of an ipsilateral femoral hernia was detected (Figure 3A). Subclinical contralateral coexistence of obturator hernia (left-sided obturator hernia) was not found. The peritoneum was incised to dissect the preperitoneal space, and a peritoneal flap was made both ventrally and dorsally. The dorsal peritoneal flap was extended wider than the usual inguinal hernia procedure in order to confirm the obturator canal orifice, which exists in the dorsal and medial parts of the myopectineal orifice. The tag of fat tissue and the hernial sac were teased out from the narrow canal for the prophylaxis of the neuralgia caused by the obturator nerve compression by this fat plug (Figure 3B) [2]. The dissected preperitoneal space measured approximately 16 × 11 cm. We adapted Bard Soft Mesh (15 × 10 cm) (Becton Dickinson, Franklin Lakes, NJ, USA) by cutting the four corners to ensure better shape-fitting. Only one artificial mesh (rectangular shape) was placed to cover all four hernia orifices and fixed with metal spiral tackers (seven shots in total). Three of the seven shots were fired into the pubic bone (Cooper’s ligament). One shot was fired into the pubic tubercle near the midline, and the other two shots were fired just next to the obturator canal orifice in order to cover the obturator canal orifice surely and firmly (Figure 3C). The peritoneum was closed by a continuous suture using an absorbable barbed suture (3-0 V-Loc PBT; Covidien/Medtronic, Minneapolis, MN, USA).

Her recovery was rather slow because of the weakness due to old age and diabetes mellitus. She was uneventfully discharged home on postoperative day 14 and developed no hernia recurrence for three years after surgery.

## Discussion

The obturator foramen is the largest foramen in the human body. It is covered by a strong fibrous membrane called the obturator membrane. The obturator canal is a small and narrow passageway in the superior aspect of the obturator membrane (approximately 0.3 cm wide and 3.0 cm long) through which the obturator nerve, artery, and vein pass. An obturator hernia is defined as herniation of the abdominal contents through the obturator canal toward the thigh.

Prior to the invention of CT, a definitive diagnosis of obturator hernia was generally difficult to achieve because of the lack of identifiable physical findings. A bulge or lumps due to the obturator hernia can not often be palpated on the body surface. Therefore, late diagnosis may necessitate the resection of the necrotic intestine by emergency surgery and may lead to a poor prognosis.

In the past, the mortality rate for patients undergoing emergency operations (open surgeries) was as high as 40% [1]. But nowadays, the mortality rate is 3% in emergency laparoscopic surgeries [4]. In emergency laparotomy, closure of the obturator canal tends to be inadequate or excluded because saving the lives of patients was the primary concern of the surgeons. Therefore, surgeons concentrated on relieving the ileus, removing necrosis, and reconstructing the bowel. In high-risk surgeries, some surgeons may have little inclination to close the hernia orifice. Inadequate orifice closure may result from simple sewing of the peritoneum around the hernia orifice or suturing of nearby organs (uterus or broad ligament of the uterus) onto the canal. Simple sewing leaves a recessed canal hole, and the organ stitches can sometimes become detached. Hence, the incarceration may recur postoperatively.

**Figure 4:**
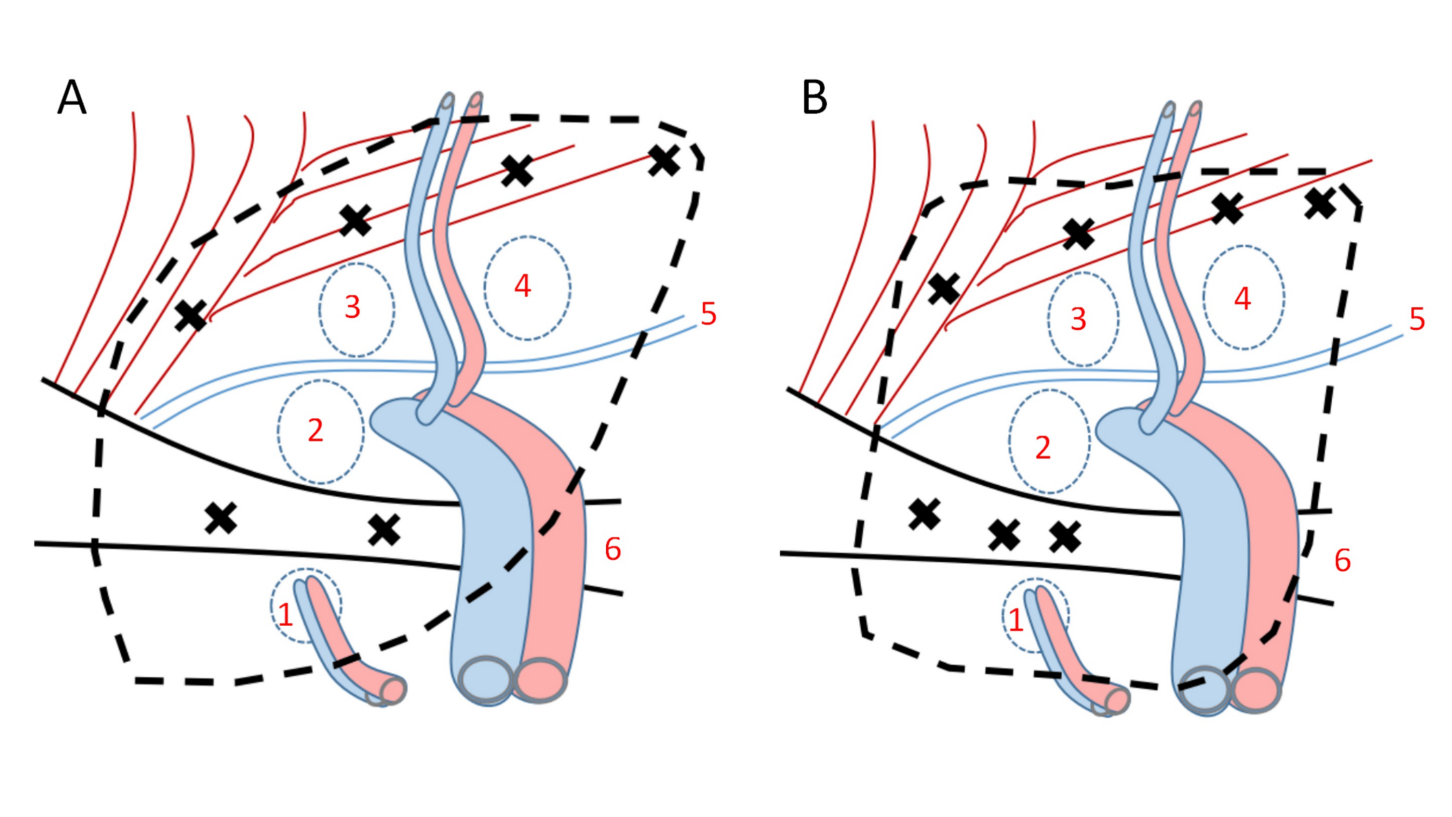
Complete and simultaneous coverage of all four hernia orifices with a single large mesh Two methods can be used to cover both the obturator canal and myopectineal orifice: overlapping two meshes or using only one large mesh. There are also two methods to cover all four hernia orifices using a single large mesh: (A) oblique placement of leaf-shaped sheet of the Bard 3D Max (light type, large size, 15.7 × 10.3 cm) or (B) using one rectangular sheet of Bard Soft Mesh (15 × 10 cm) without any slanting. The latter approach was used in the present case. Image credits: Shogo Maruzen The locations where the tackers were placed are denoted by x. The black dotted lines are the area covered by a single large mesh. 1, obturator canal orifice; 2, femoral hernia orifice; 3, internal inguinal hernia orifice; 4, external inguinal hernia orifice; 5, iliopubic tract; 6, Cooper’s ligament (the pubic bone)

Obturator hernias are concurrently associated with other types of groin hernias, such as femoral hernias, internal inguinal hernias, and external inguinal hernias [4]. Therefore, it is ideal to prevent other hernias simultaneously rather than only fix the obturator hernia. In other words, both the obturator canal and myopectineal orifice should be covered at the same time. Two methods can be used to cover all four orifices: overlapping two meshes [5] or using only one large mesh. Repairing with only one large mesh is more simple and easier than overlapping two mashes. Additionally, there are two ways in which a single large mesh can be used. The first involves oblique placement of a leaf-shaped sheet of the Bard 3D Max (light type, large size, 15.7 × 10.3 cm) [6], and the second involves placement of one rectangular sheet of the Bard Soft Mesh (15 × 10 cm) without any slanting. The latter approach was used in the present case (Figures 4A, 4B). If there is only an obturator hernia and no other hernias, we will simultaneously cover both the obturator canal and the myopectineal orifice. However, in case of very dangerous patients, we will cover only the obturator canal.

Patients with an obturator hernia are usually asymptomatic when the intestine is not present within the canal. Generally, however, when the intestine is within the canal, patients experience pain from the thigh to the hip joint. Occasionally, patients do not develop abdominal pain or nausea because Richter-type herniation involves partial obstruction of the anti-mesenteric portion of the bowel. The bowel contents can somehow pass through the narrow segment despite incarceration. Therefore, novice general surgeons may not be able to consider an obturator hernia, and orthopedic surgeons may mistake it for sciatica [7]. Gastrointestinal surgeons do not necessarily have a higher accurate diagnosis rate compared to other emergency physicians or general internists. This is because the diagnosis of obturator hernia is difficult even for gastrointestinal surgeons.

On the other hand, the Howship-Romberg sign manifests as pain in the medial and upper sides of the thigh secondary to obturator nerve irritation. Mildly affected patients experience only numbness or dull pain, whereas severely affected patients develop tremendous pain (shooting or burning pain). The pain is more intense, with extension, abduction, and internal rotation of the hip joint. The obturator nerve is irritated by the incarcerated intestine in such cases. The incarceration can be released by pushing it back into the abdominal cavity with an echo probe manipulation while observing it; alternatively, the lower extremity can be moved to release the incarceration. In some cases, a combination of both methods is used [8,9]. Once the incarcerated hernia is released, the patient’s symptoms are relieved, and the possibility of necrosis is reduced. In patients with necrosis or perforation, artificial prosthesis insertion is not commonly performed because of the risk of mesh infection. We previously experienced a case in which a femoral abscess formed postoperatively despite the fact that infection control had been meticulously performed by suturing the fallopian tube onto the obturator canal instead of using an artificial mesh, following thorough saline washing of the hernial cavity [10]. The abscess healed with percutaneous drainage of the thigh. Temporal release from incarceration and performing a hernia repair using mesh in an elective operation was an effective, comprehensive treatment for the present case.

## Conclusions

This report presents a clinically notable case of laparoscopic obturator hernia repair following the proper release of intestinal incarceration. Reports of cases in which a single mesh sheet was used to cover all four groin hernia orifices are scarce. Complete and simultaneous coverage of the obturator hernia and myopectineal orifice with one large rectangular mesh is an efficient obturator hernia repair technique.
